# Recurrent invasive liver abscess syndrome induced by *Klebsiella pneumoniae* with emerging drug resistance: a case report and literature review

**DOI:** 10.3389/fmed.2025.1605284

**Published:** 2025-07-30

**Authors:** Yongmei Li, Zheng He, Fengjiao Wu, Jialin Li, Chengcheng Wang

**Affiliations:** ^1^Department of Clinical Pharmacy, Chengyang People’s Hospital, Qingdao, China; ^2^Department of Neurosurgery, Qilu Hospital (Qingdao), Cheeloo College of Medicine, Shandong University, Qingdao, China; ^3^Department of Pharmacy, Qilu Hospital (Qingdao), Cheeloo College of Medicine, Shandong University, Qingdao, China

**Keywords:** invasive liver abscess syndrome, emerging drug resistance, brain abscess, *Klebsiella pneumoniae*, case report

## Abstract

**Background:**

Invasive liver abscess syndrome (ILAS) caused by hypervirulent *Klebsiella pneumoniae* (hvKp) is a life-threatening infection associated with high mortality, particularly when complicated by brain abscesses. The emergence of carbapenem-resistant hypervirulent *K. pneumoniae* (CR-hvKp) during treatment, driven by emerging resistance, poses significant therapeutic challenges.

**Case presentation:**

We describe a 70-year-old diabetic male with recurrent ILAS who developed sequential multi-organ infections, including urinary tract infection, bacteremia, lung abscess, and brain abscess. Initial isolates were identified as carbapenem-susceptible; however, under prolonged antimicrobial pressure, they were transformed into CR-hvKp carrying the KPC gene. Treatment escalated from meropenem to ceftazidime-avibactam plus tigecycline, resulting in clinical improvement and discharge after 48 days.

**Conclusion:**

This case highlights the critical challenge of emerging resistance in hvKp during prolonged therapy, emphasizing the need for early molecular surveillance, multidisciplinary collaboration, and optimized antibiotic stewardship to mitigate treatment failure in ILAS.

## Introduction

Invasive liver abscess syndrome (ILAS), caused by hypervirulent *Klebsiella pneumoniae* (hvKp), is a severe systemic infection characterized by liver abscess formation and metastatic spread to organs such as the lungs, eyes, and central nervous system (CNS) (1). Initially reported in Taiwan, the global incidence of ILAS has risen steadily in recent years, with a notably higher prevalence among diabetic patients. ILAS can lead to a series of serious complications, including bacteremia, lung abscess, metastatic endophthalmitis, and brain abscess, all of which pose a significant threat to patient survival. Among these, brain abscess stands out as a particularly lethal complication, with mortality rates ranging from 48.5 to 53% in ILAS patients (2, 3), despite advances in imaging and antimicrobial therapy (4). Prognosis is especially poor in cases involving multiple organ systems, septic shock, or significant underlying comorbidities. The management of ILAS necessitates a multidisciplinary approach, integrating organ support, prolonged antibiotic therapy, and surgical intervention; however, clinical outcomes remain unpredictable. Historically, hvKp strains exhibited susceptibility to most antibiotics, but the emergence of carbapenem-resistant hvKp (CR-hvKp) has introduced a troubling convergence of hypervirulence and multidrug resistance, presenting a formidable global health challenge (5). The emergence of CR-hvKp markedly complicates ILAS treatment, particularly in cases of systemic dissemination, such as those involving brain abscesses.

In this report, we describe a case of recurrent ILAS in a patient who experienced sequential infections across multiple organs, including the urinary tract, bloodstream, lungs, and brain, over the course of the disease. The pathogen exhibited a dynamic shift from a susceptible phenotype to carbapenem resistance. Through this case study and a comprehensive literature review, we examine the patient’s clinical presentation, diagnostic workup, therapeutic interventions, and prognosis. Our aim is to identify effective strategies for managing such complex infections and to provide clinicians with actionable insights to enhance the recognition and treatment of similar cases.

## Case presentation

A 70-year-old Han Chinese male with a history of type 2 diabetes and hypertension presented to the emergency department on December 6, 2024. Eighteen months prior, he had undergone two percutaneous drainage procedures for liver abscesses, with pus and blood cultures confirming susceptible *Klebsiella pneumoniae*. The patient reported a 3-day history of fever (peak 39.5°C), chills, cough, and diarrhea. Upon admission, he exhibited acute-onset lower limb weakness, dysarthria, and lethargy. Physical examination revealed a temperature of 37.3°C, heart rate of 112 beats/min, respiratory rate of 32 breaths/min, and blood pressure of 149/75 mmHg, and bilateral lung rales, with a Glasgow Coma Scale (GCS) score of 12 (E3V4M5).

**Figure 1:**
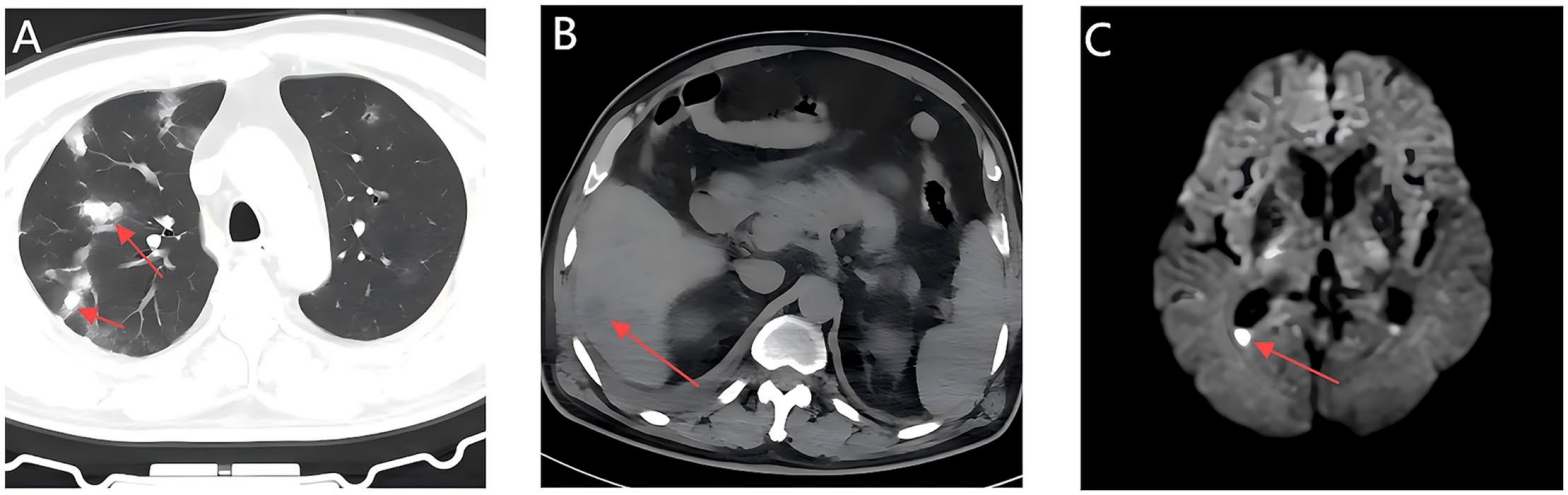
Initial multimodal imaging findings within 24 hours of admission; Chest CT, Abdominal CT, and Brain MRI. **(A)** A Chest CT scan shows multiple patchy hyperdense opacities in bilateral lungs. **(B)** An abdominal CT scan shows hypodense lesion in the right posterior hepatic lobe (arrow). **(C)** Axial MRI head shows mild enlargement of the supratentorial ventricular system, with fluid levels in the occipital horn of the lateral ventricle accompanied by hyperintensity on DWI (arrows).

Laboratory results showed a white blood cell count of 7.66 × 10^9^ /L (reference range: 3.5–9.5 × 10^9^ /L), thrombocytopenia with a platelet count of 37 × 10^9^ /L (reference range: 125–350 × 10^9^ /L), elevated high-sensitivity C-reactive protein of 236.62 mg/L (reference range:0–10 mg/L), hyperglycemia with a glucose level of 20.3 mmol/L (reference range: 4.1–5.9 mmol/L), glycated hemoglobin of 6.3% (reference range: 3.8–5.8%), procalcitonin of 42.86 ng/mL(reference range: 0–0.5 ng/mL), and creatinine of 229 μmol/L (reference range: 62–115 μmol/L). Urinalysis indicated nitrite positivity and a bacterial count of 48,783.8 U/L (reference range: 0–10 U/L). Multimodal imaging revealed bilateral pulmonary infiltrates (Figure 1A), a hepatic abscess (Figure 1B), mild ventricular enlargement with diffusion-weighted imaging (DWI) hyperintensity (Figure 1C), and a heterogeneous hepatic lesion (5.4 cm × 3.4 cm) in the right posterior lobe on ultrasound. Initial diagnoses included sepsis, severe pneumonia, multi-organ dysfunction syndrome (MODS), acute pyelonephritis, liver abscess, type 2 diabetes, and thrombocytopenia.

**Figure 2:**
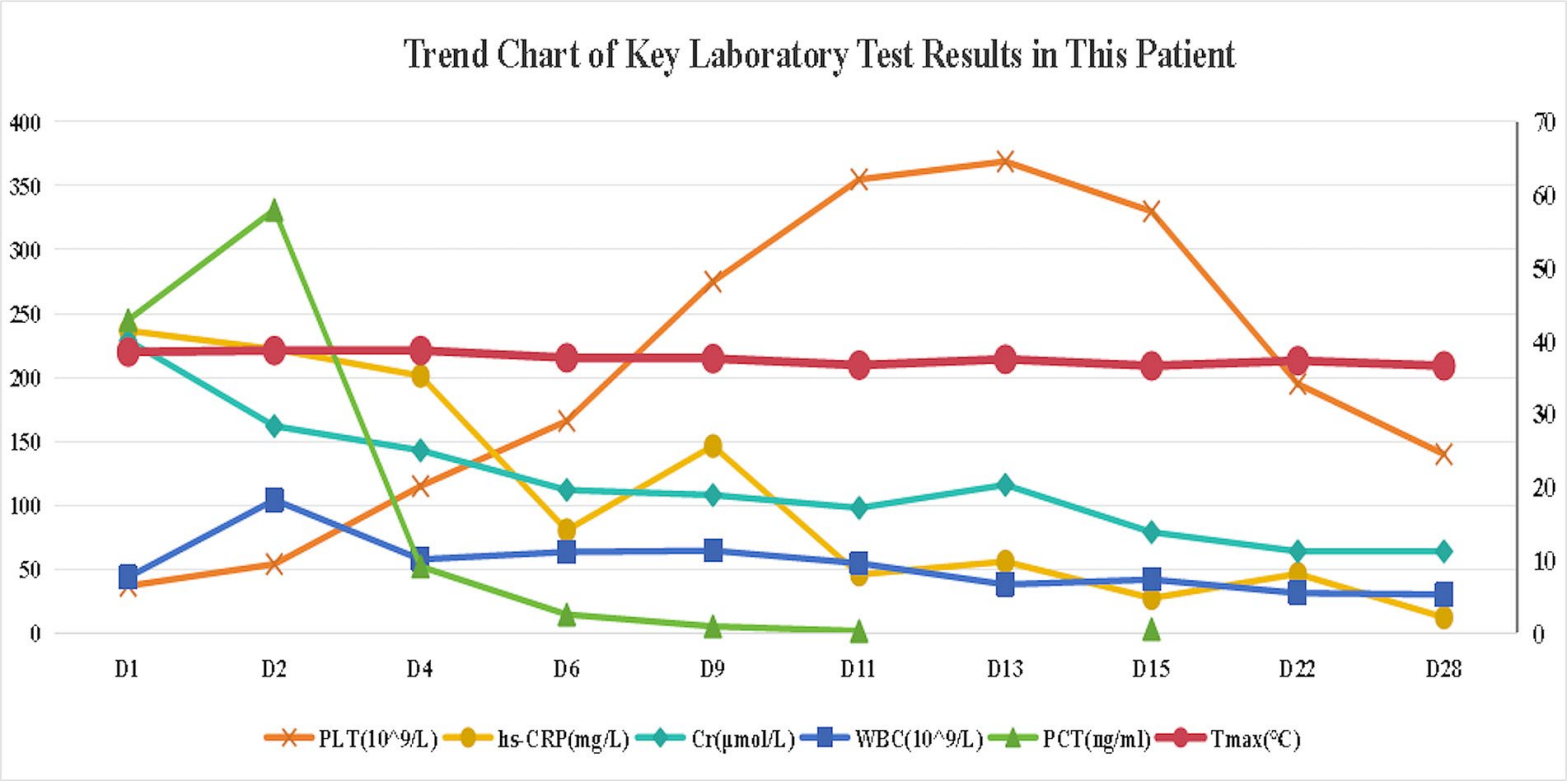
Dynamic changes in key biomarkers following hospital admission.

Empiric meropenem (1 g q12h) was initiated for infection control, alongside insulin pump therapy (insulin aspart) for glycemic management. Neurological symptoms improved within hours, with the patient regaining clear consciousness, normal speech, and muscle tone. On day 2, bilateral blood cultures indicated critical Gram-negative rod values, and procalcitonin levels rose (Figure 2). By day 3, urine, blood, and sputum cultures confirmed ESBL-negative *K. pneumoniae* (Table 1), while high-throughput sequencing of blood revealed *K. pneumoniae* (43,033 reads per Million), with sequencing identifying *SHV* (79 reads) and *iroN* (30 reads) genes (Table 2), suggesting ILAS. Meropenem was escalated to 1 g every 8 h as lung abscesses worsened. Laboratory markers, including infection indices and renal function, subsequently improved (Figure 2).

**Table 1:** Antimicrobial susceptibility profiles of *K. pneumoniae* isolates from multiple sites (Sensitive vs. Resistant strains).

Antimicrobial agent	Blood (D1)	Urine (D1)	Sputum (Sensitive, D1)	Pus (D16)	Sputum (Resistant, D7)	Sputum (Resistant, D8)	Sputum (Resistant, D21/D27)
Ampicillin/Sulbactam	4 (S)	4 (S)	8 (S)	≤8 (S)	≥32 (R)	≥32 (R)	≥32 (R)
Piperacillin/Tazobactam	≤4 (S)	≤4 (S)	≤4 (S)	≤8 (S)	≥128 (R)	≥128 (R)	≥64 (R)
Cefuroxime	22^a^ (I)	23^a^ (S)	23^a^ (S)	≤4	6 (R)	6 (R)	>16 (R)
Ceftriaxone	≤1 (S)	≤1 (S)	≤1 (S)	-	≥64 (R)	≥64 (R)	-
Aztreonam	≤1 (S)	≤1 (S)	≤1 (S)	≤4 (S)	≥64 (R)	≥64 (R)	>16 (R)
Imipenem	≤1 (S)	≤1 (S)	≤1 (S)	0.5 (S)	≥16 (R)	≥16 (R)	>16 (R)
Amikacin	≤2 (S)	≤2 (S)	≤2 (S)	≤16 (S)	≥64 (R)	≥64 (R)	>64 (R)
Levofloxacin	≤0.25 (S)	≤0.25 (S)	≤0.25 (S)	≤0.12 (S)	≥8 (R)	≥8 (R)	>8 (R)
Meropenem	29^a^ (S)	30^a^ (S)	28^a^ (S)	≤0.06 (S)	12^a^ (R)	10^a^ (R)	>16 (R)
Ceftazidime/Avibactam	-	-	-	≤0.5 (S)	20^a^ (R, misclassified)	20^a^ (R, misclassified)	4 (S)
Tigecycline	-	-	-	0.5 (S)	≥8 (R)	≥8 (R)	4 (I)

S, Susceptible; I, Intermediate; R, Resistant. MIC values are expressed in μg/mL, Results annotated with “a” indicate results obtained by Kirby-Bauer disk diffusion method. “misclassified” indicates initial misinterpretation corrected by MIC testing. “-” indicates not tested or data unavailable.

**Table 2:** Summary of pathogen genomic analysis results.

Hospital day	Sample type	Microbial type	Result metric	Detected genes
D3	Blood	*K. pneumoniae*	43,033 reads per Million	*SHV, iroN*
D12	Sputum	*K. pneumoniae*	Ct 17.42	*KPC*
D15	CSF	*K. pneumoniae*	1,124 reads	Salmochelin, Aerobactin

Primary Y-axis (Left): Platelet count (PLT, ×10^9^ /L), C-reactive protein (CRP, mg/L), and serum creatinine (Cr, μmol/L), Secondary Y-axis (Right): White blood cell count (WBC, ×10^9^ /L), procalcitonin (PCT, ng/mL), and maximum daily temperature (Tmax, °C).

**Figure 3:**
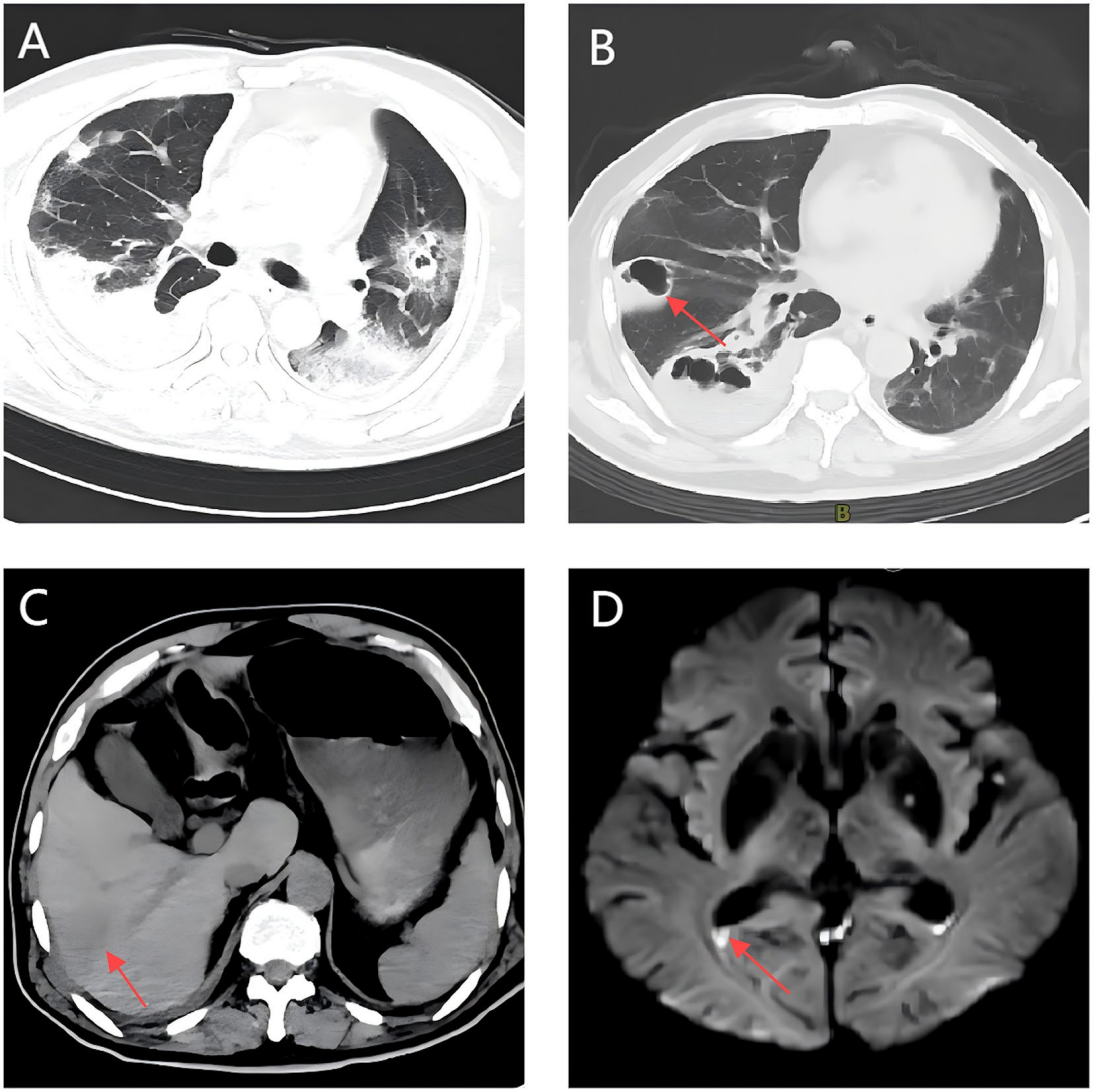
Follow-up multimodal imaging findings during treatment: Chest CT, Abdominal CT, and Brain MRI. **(A)** A Chest CT scan shows bilateral cavitary pneumonia with right pleural effusion. **(B)** Multiple thin-walled pulmonary abscesses with rim enhancement (arrow). **(C)** Hepatic abscess (segment 6) with hepatic vein thrombosis (arrow). **(D)** Bilateral ring-enhancing cerebral lesions with DWI hyperintensity in ventricular posterior horns (arrow).

Persistent fever occurred during hospitalization (Figure 2). On day 7, chest CT revealed bilateral patchy and nodular opacities with cavities and increased right pleural effusion (Figure 3A), with two non-consecutive sputum cultures indicating pan-drug-resistant *K. pneumoniae* (Table 1). On day 12, molecular testing of sputum detected the *KPC* gene (Ct 17.42). Initial Kirby-Bauer disk diffusion testing suggested resistance to ceftazidime-avibactam (20-mm inhibition zone), but subsequent broth microdilution testing confirmed susceptibility (MIC 2 μg/mL), prompting a switch to ceftazidime-avibactam (2.5 g q8h) to target the emerging *KPC*-producing isolate. However, fever persisted with new-onset somnolence and delayed responses. On day 15, imaging showed multiple high-density, thin-walled lung abscesses (Figure 3B), a hypodense lesion in liver segment S6 (Figure 3C), and brain abscesses with ventricular empyema (Figure 3D). The patient was transferred to another facility, where lumbar puncture and CSF sequencing confirmed *K. pneumoniae* infection, with virulence gene salmochelin (6 reads) and aerobactin (2 reads). Due to CNS progression, limited ceftazidime-avibactam CNS penetration, and high cost, treatment was adjusted to meropenem (2 g q8h) plus tigecycline (50 mg q12h) to enhance CNS penetration and manage persistent infection, despite known *KPC* resistance in sputum samples. On day 16, ultrasound-guided aspiration of the liver abscess yielded white, viscous pus, culturing susceptible *K. pneumoniae* (Table 1). Post-treatment, neurological symptoms markedly improved, fever resolved, and cough and sputum production decreased, with infection markers trending toward normal. On day 25, sputum culture revealed *KPC*-producing carbapenem-resistant *K. pneumoniae* (CRKP) (Table 1). Based on the patient’s improved CNS symptoms, the persistence of CRKP in sputum cultures, and the shift in tigecycline susceptibility, the regimen was adjusted to tigecycline (100 mg q12h) and ceftazidime-avibactam (2.5 g q8h) to optimize coverage against persistent pulmonary infection. Symptoms stabilized, and follow-up imaging indicated significant resolution of lung abscesses, reduced cerebral abscesses, and near-complete clearance of ventricular empyema. On day 32, gastrointestinal bleeding occurred, accompanied by hemoglobin drop to 53 g/L, which was managed with blood transfusion, esomeprazole, thrombin powder, and octreotide. Bleeding resolved by day 48 of hospitalization, and the patient was discharged on January 23, 2025, with sustained clinical improvement.

## Discussion

This case report presents a patient with recurrent ILAS caused by hvKp. During the disease course, the patient developed sequential multiorgan infections, including urinary tract infection, bloodstream infection, lung abscess, and brain abscess, with the pathogen exhibiting emerging resistance from a susceptible phenotype to carbapenem resistance. This case underscores the highly invasive nature of hvKp infections and highlights the progressive emergence of drug resistance during prolonged, multi-phase antibacterial therapy, which significantly complicates treatment decisions. These observations have critical implications for contemporary anti-infective strategies.

Following intestinal colonization, hvKp breaches the mucosal barrier, invades the liver via the portal vein, and forms abscesses. These hepatic abscesses may trigger hematogenous dissemination, causing severe extrahepatic infections, including brain abscesses, purulent meningitis, endophthalmitis, and necrotizing fasciitis. The coexistence of these complications with hvKp-induced liver abscesses defines ILAS (1). The hypervirulence of hvKp is driven by key virulence factors, notably the polysaccharide capsule (K1/K2 serotypes) and siderophore systems (6). The capsule shields hvKp from phagocytosis by polymorphonuclear leukocytes, promoting immune evasion and tissue invasion, and siderophores—such as aerobactin (encoded by *iucABCD-iutA*) and salmochelin (encoded by *iroBCDN*)—enhance iron acquisition and adaptability (7). These siderophores are expressed at markedly higher levels in hvKp compared to classical *K. pneumoniae* (cKp) (8). Metagenomic sequencing has revealed that most hvKp strains carry a plasmid *pLVPK* containing four major virulence genes: *iuc* (aerobactin biosynthesis), *rmpA* and *rmpA2* (capsule production regulators), and *iro* (salmochelin biosynthesis). These genes are pivotal to hvKp hypervirulence and may serve as reliable molecular markers for its identification (9).

Ethnic background (Asian populations), diabetes, and the use of specific drugs such as ampicillin or proton pump inhibitors are the primary risk factors for ILAS (9). Diabetic patients are particularly vulnerable due to impaired mucosal integrity, enhanced bacterial translocation, immune dysregulation, and microthrombosis (10). The patient in this case exhibited several of these risk factors, including Asian ethnicity and diabetes mellitus, and had a history of liver abscesses requiring percutaneous drainage. During the disease progression, the patient experienced two episodes of bloodstream infection, followed by subsequent metastatic infections such as lung abscess and brain abscess. Genomic sequencing identified the *iroN*, salmochelin, and aerobactin gene, and both clinical manifestations and genetic test results strongly supported a diagnosis of ILAS caused by hvKp.

Traditionally, hvKp has been regarded as highly susceptible to clinically used antibiotics, except for ampicillin, despite its hypervirulent phenotype (11). However, the growing spread of mobile genetic elements carrying resistance genes has fueled an increase in CR-hvKp, a strain that merges high virulence with multidrug resistance, posing substantial therapeutic challenges. Two evolutionary pathways for resistance development in hvKp have been proposed: hvKp may acquire resistance plasmids from cKp, conferring associated resistance, or cKp may obtain virulence plasmids from hvKp, resulting in both hypervirulence and multidrug resistance (12, 13). The primary resistance mechanisms include: (1) Extended-spectrum *β*-lactamase (ESBL)-mediated resistance: ESBLs, classified into *TEM, SHV*, *CTX-M*, and *OXA* types based on plasmid gene homology. Notably, *CTX-M* is the predominant ESBL genotype in hvKp isolates from China (14). (2) AmpC *β*-lactamase-mediated resistance: *AmpC,* another critical *β*-lactamase produced by Gram-negative bacilli, hydrolyzes cephamycins (e.g., cefoxitin and cefotetan) when overexpressed, further amplifying resistance. (3) Carbapenem resistance: The principal mechanism of carbapenem resistance in *K. pneumoniae* involves the production of carbapenemases, such as *KPC*, New Delhi metallo-*β*-lactamase 1 (*NDM-1*), and *OXA-48*. In China, *KPC*-2 accounts for 94.4% of carbapenemases identified in CR-hvKp isolates (11). Additional mechanisms, including outer membrane protein loss, efflux pump overexpression, and biofilm formation, also contribute significantly to hvKp resistance (7). In this case, initial cultures indicated carbapenem-susceptible *K. pneumoniae*, but sequencing on day 2 identified the *SHV* gene, potentially indicating intrinsic ampicillin resistance (*SHV*-1) (15) or an emerging variant, though limited sequencing resolution precluded definitive phenotyping. By day 6 of meropenem therapy, sputum cultures identified CRKP with the *KPC* gene, initially misclassified as resistant to ceftazidime-avibactam (20-mm zone by disk diffusion). Per CLSI M100 (32nd edition) (16), isolates with 20–22 mm inhibition zones require MIC confirmation to rule out false susceptibility or resistance; subsequent broth microdilution testing confirmed susceptible with an MIC of 2 μg/mL (17). It may stem from mutations under antibiotic pressure or horizontal plasmid transfer, potentially enabled by genetic mechanisms integrating resistance and virulence traits (18). The concurrent case of carbapenem-resistant *Escherichia coli* was identified in the same ward raises the possibility of nosocomial transmission. However, due to limitations in laboratory capabilities—such as the lack of homology sequencing, whole-genome sequencing, or plasmid tracing—this study could not fully elucidate the *in vivo* evolutionary dynamics of the bacteria or provide definitive conclusions. These gaps highlight critical directions for future research.

CNS involvement occurs in 2–8% of ILAS cases, manifesting as meningitis, brain abscesses, or ventriculitis (1, 19). The development of brain abscesses is not only associated with the high invasiveness of hvKp but may also be closely linked to compromised host immunity, hematogenous dissemination of the pathogen, and disruption of the blood–brain barrier (20). Fever, altered consciousness, and seizures are the most common clinical presentations (2). Once CNS infection occurs, mortality rates can reach 48.5–53% (2, 3), with CR-hvKp-linked intracranial infections approaching 100% fatality in some reports (21). Literature indicates that ILAS-related brain abscesses predominantly affect Asian males (80%) with diabetes (80%), presenting as multifocal lesions in the basal ganglia, cerebellum, and frontal lobes, and prognosis is notably worse in patients with drug-resistant infections, multifocal abscesses, or concurrent multi-organ failure. Here, the patient’s initial neurological symptoms (somnolence, dysarthria) resolved with meropenem but recurred with abscess progression, possibly due to fluctuating antibiotic penetration across the blood–brain barrier—higher during early inflammation and reduced with encapsulation. Despite the high mortality associated with hvKp-linked intracranial infections, the patient’s survival can be attributed to several factors. Firstly, the intracranial infection may have involved a susceptible hvKp strain, as suggested by susceptible isolates in pus (Table 1, Day 16). Additionally, multidisciplinary support, along with adequate antibiotic dosing, nutritional support, and effective glycemic control, likely contributed to the favorable outcome. Notably, such transient improvement in early hospitalization may, however, mislead clinical assessment, underscoring the need to include brain abscess in the differential diagnosis and strongly recommend close monitoring of imaging changes.

Antibiotics are a cornerstone of ILAS treatment. HvKp typically responds to third-generation cephalosporins, quinolones, and carbapenems, with cephalosporins favored for initial treatment (1). When infections extend beyond the liver, tissue penetration of antibiotics must be considered based on the site of dissemination. For brain abscess management, antibiotics should possess specific properties: activity against pathogens in the typically acidic abscess environment, adequate penetration into the abscess cavity, low risk of inducing resistance, and minimal toxicity (22). Cefotaxime and ceftriaxone, with their high cerebrospinal fluid (CSF) penetration, are recommended as first-line treatments for Enterobacterales-induced brain abscesses (23). Conversely, CRKP infections offer limited options. According to IDSA guidelines (24), preferred treatments for CRKP include meropenem-vaborbactam, ceftazidime-avibactam, and imipenem-cilastatin/relebactam. Tigecycline serves as an alternative recommended when *β*-lactam agents are ineffective or poorly tolerated. In this case, the patient initially received meropenem for infection control, resulting in improved infection markers. However, progression of pulmonary infection and the emergence of CR-hvKP in sputum cultures necessitated a switch to ceftazidime-avibactam. A hospital transfer disrupted continuity; later, meropenem plus tigecycline improved brain abscesses, though sputum remained CR-hvKP-positive. A subsequent regimen of tigecycline (100 mg q12h) and ceftazidime-avibactam (2.5 g q8h) stabilized the patient’s condition. Notably, current guidelines do not support combination antibiotic therapy for CRKP infections (24). Excessive combination has been associated with potential adverse reactions, as seen in the patient’s subsequent gastrointestinal bleeding, with prothrombin time (PT) elevated to 16.8 s, possibly related to tigecycline use due to impaired vitamin K metabolism. These findings underscore the ongoing challenge of devising rational antibiotic regimens for such cases.

Liver abscess recurrence caused by hvKp is rarely reported (25, 26). Studies based on small cohorts of recurrent cases indicate an average interval between initial onset and recurrence of 7.6 years (range: 2–20 years) (27–29). Poor glycemic control, K1 capsular serotype (27, 28), and ESBL production (29) are identified as primary risk factors for recurrence. Potential mechanisms underlying recurrence include incomplete clearance of infection foci, inadequate antibiotic penetration, and biofilm formation. In this case, the patient experienced chills and high fever one month post-initial liver abscess drainage, with a new abscess in segment S7 (7.2 cm × 8.1 cm) and reduction of the original abscess (8.1 cm × 4.7 cm to 3.2 cm × 3.3 cm), suggesting relapse due to insufficient treatment. A second recurrence occurred 1.5 years after subsequent drainage, underscoring the significant burden of repeated episodes on the patient. For individuals at high risk of recurrence, we recommend aggressive surgical interventions (e.g., multiple drainage procedures), extended antibiotic therapy, strict glycemic control, and regular imaging follow-up to monitor disease progression.

### Limitations and future research directions

This study, being a single-case report, is subject to potential selection bias. The absence of whole-genome sequencing limits the detailed characterization of resistance mechanisms. Future multicenter studies are warranted to elucidate the emerging resistance mechanisms of CR-hvKp, optimize therapeutic approaches, and improve ILAS prognosis.

## Conclusion

This case highlights the critical need for monitoring resistance evolution in recurrent ILAS, necessitating dynamic adjustments to antibiotic regimens and multidisciplinary management to enhance patient outcomes. Future studies must prioritize investigating the epidemiology and optimizing treatment strategies for CR-hvKp infections.

## Data Availability

The datasets presented in this study can be found in online repositories. The names of the repository/repositories and accession number(s) can be found in the article/supplementary material.
